# Disseminated Extrapulmonary Tuberculosis and Pulmonary Mycobacterium avium Complex Co-infection in a Newly Diagnosed HIV Patient: A Case Report

**DOI:** 10.7759/cureus.91532

**Published:** 2025-09-03

**Authors:** Priscila Lopez, Wajid Ali, Webster Donaldy, Gurveer Singh Raien, Vel Sivapalan

**Affiliations:** 1 Internal Medicine/Infectious Diseases, Harlem Hospital Center, New York, USA; 2 Internal Medicine, Harlem Hospital Center, New York, USA; 3 Internal Medicine, New York City (NYC) Health and Hospitals/Harlem, New York, USA; 4 Infectious Diseases, Harlem Hospital Center, New York, USA

**Keywords:** disseminated mycobacterium, hiv, mycobacterial lymphadenitis, mycobacterium, opportunistic infections, tuberculosis

## Abstract

Disseminated extrapulmonary tuberculosis (TB) and *Mycobacterium avium* complex (MAC) are both serious opportunistic infections (OIs), commonly encountered in immunocompromised individuals, particularly those with HIV. Co-infection with both pathogens is rare but can present significant diagnostic and therapeutic challenges.

We report the case of a 45-year-old male who was newly diagnosed with HIV infection and who developed disseminated extrapulmonary TB alongside pulmonary MAC, highlighting the complexities of diagnosis, treatment, and management in the context of dual mycobacterial infections. This case underscores the importance of early recognition and coordinated care in managing multi-pathogen infections in immunocompromised patients.

## Introduction

Opportunistic infections (OIs) are a leading cause of mortality in patients with acquired immunodeficiency syndrome (AIDS). The human immunodeficiency virus (HIV) pandemic has worsened the situation, not only by leading to the resurgence of tuberculosis (TB), but also by suppressing the host immune system, which provides an opportunity for infection by non-TB mycobacteria (NTM) [1].

TB, caused by *Mycobacterium tuberculosis* (MTB), is primarily a respiratory infection, but up to 15% of cases worldwide involve extrapulmonary sites, complicating both diagnosis and management, as well as posing a public health risk [2-4].

The incidence of TB in the United States has decreased over the years, but despite the low incidence, detection remains important because, if not recognized, it can progress to disseminated TB, which is a progressive and life-threatening disease [2].

In contrast, *Mycobacterium avium* and *Mycobacterium intracellulare* are classified as nontuberculous mycobacterial pathogens, frequently grouped together as *Mycobacterium avium-intracellulare* (MAI) or *Mycobacterium avium* complex (MAC). MAC has become one of the most reported opportunistic bacterial infections in patients with AIDS, occurring with an annual frequency of 10%-20% [5-7]. In the United States, the incidence of NTM has risen dramatically since the 1990s [8].

In people living with HIV, pulmonary-limited MAC is unusual; most cases manifest as disseminated infection [9]. NTM infections are usually seen when the HIV CD4 count is less than 50 cells/mm³; however, there is no statistically significant difference in the incidence of NTM infections among different baseline viral loads [1].

At-risk populations for MTB include the elderly, immunocompromised individuals, especially those with HIV; the unhoused; individuals with excessive alcohol use; and immigrants from areas with high TB rates [2,4,10].

The duration of symptoms before diagnosis is variable. Patients may experience progressive symptoms and signs such as anorexia, fatigue, dyspnea, night sweats, fever, abdominal pain, hemoptysis, headache, mental changes, pleural effusion, ascites, and lymphadenopathy over days to weeks, or occasionally over several months [11].

Disseminated extrapulmonary TB is an uncommon presentation, representing only 15%-20% of global TB cases. In cases of disseminated extrapulmonary TB, the lymphatic system is commonly affected due to the bloodborne spread of MTB [12]. When disseminated TB presents despite a normal chest radiograph, it has been described as 'cryptic tuberculosis' [11].

The diagnostic yield of lymph node biopsy or fine-needle aspiration is high. Pathologically, TB is characterized by necrotizing granulomatous inflammation of infected organs, and the yield of caseating granulomas is close to 100% [11,13]. For lymph node aspirates, Xpert MTB/RIF (rifampicin) demonstrates ~86% sensitivity (78.0 to 92.3) compared with culture, and ~99% specificity (99.1 to 99.7) using latent-class analysis [14].

The treatment regimen for patients with HIV-TB has many important considerations compared to TB cases without HIV [13]. The main treatment for disseminated extrapulmonary TB is medical, following the same principles as the treatment for pulmonary TB [12]. For drug-susceptible pulmonary TB cases with HIV infection receiving antiretroviral therapy (ART), the standard TB regimen - with two months of isoniazid (INH), RIF, pyrazinamide (PZA), and ethambutol (EMB) followed by four months of INH and RIF - is recommended. In these patients, ART should be started within the first eight weeks of beginning anti-TB treatment, and within two weeks in profoundly immunosuppressed HIV-TB patients with a low CD4 cell count (<50 cells/mm³) [13].

The recommended treatment for pulmonary MAC disease is a three-drug regimen: a macrolide (preferably azithromycin), given with EMB and RIF (RIF is preferred if drug-drug interactions or contraindications do not preclude its use). If the organism is not susceptible to a macrolide, a daily regimen with RIF, EMB, and another drug such as clofazimine, moxifloxacin, or bedaquiline is recommended. Intravenous amikacin may also be added, as improved outcomes have been shown in patients with macrolide-resistant MAC disease. The duration of treatment is based on achieving a 12-month period of negative cultures, necessitating sputum cultures every one to two months [8].

In addition, the introduction of specific combination therapies and prophylactic measures for MAC and TB, along with the availability of highly active antiretroviral therapy (HAART), has led to improved bacteriological responses and patient survival outcomes [5,7].

## Case presentation

A 45-year-old male of African descent, recently diagnosed with HIV/AIDS and hepatitis C, presented complaining of worsening abdominal pain, fevers, fatigue, anorexia, postprandial vomiting, and burning leg sensations. On admission, he was tachycardic (137 bpm), febrile (101.8°F), and hypoxic (93% on room air), with normal blood pressure and respiratory rate. He appeared cachectic, with scleral icterus, oral thrush, and diffuse, non-tender lymphadenopathy in the cervical, axillary, and inguinal regions. Abdominal exam revealed right upper quadrant (RUQ) tenderness, guarding, and hepatosplenomegaly.

**Figure 1:**
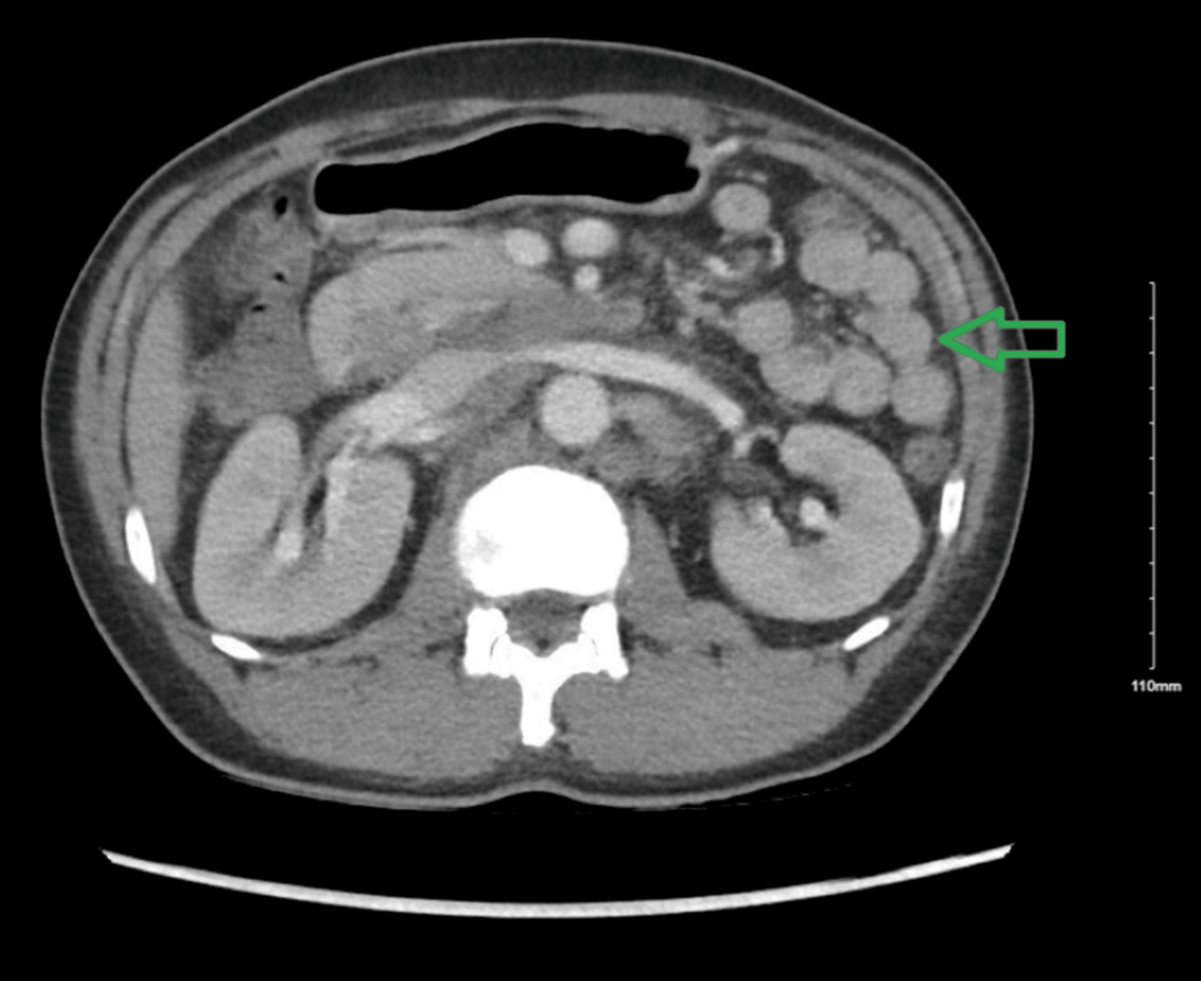
CT abdomen and pelvis without contrast (axial view) Green arrow: lymph nodes CT, Computerized Tomography

**Figure 2:**
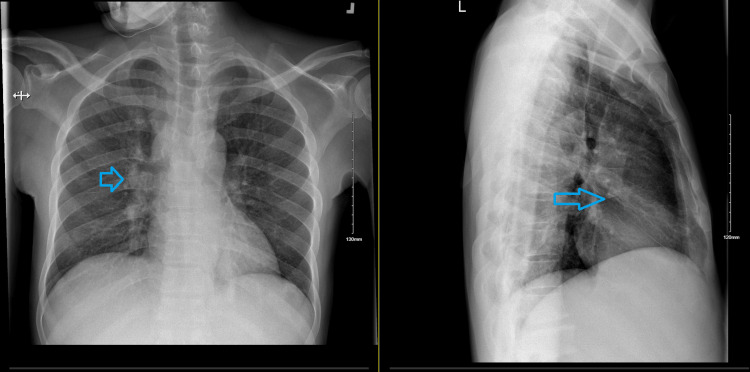
CXR (anteroposterior view - left and lateral view - right) Blue arrows: faint airspace opacity seen probably in the right middle lobe, better seen on the lateral view CXR: Chest X-ray

**Figure 3:**
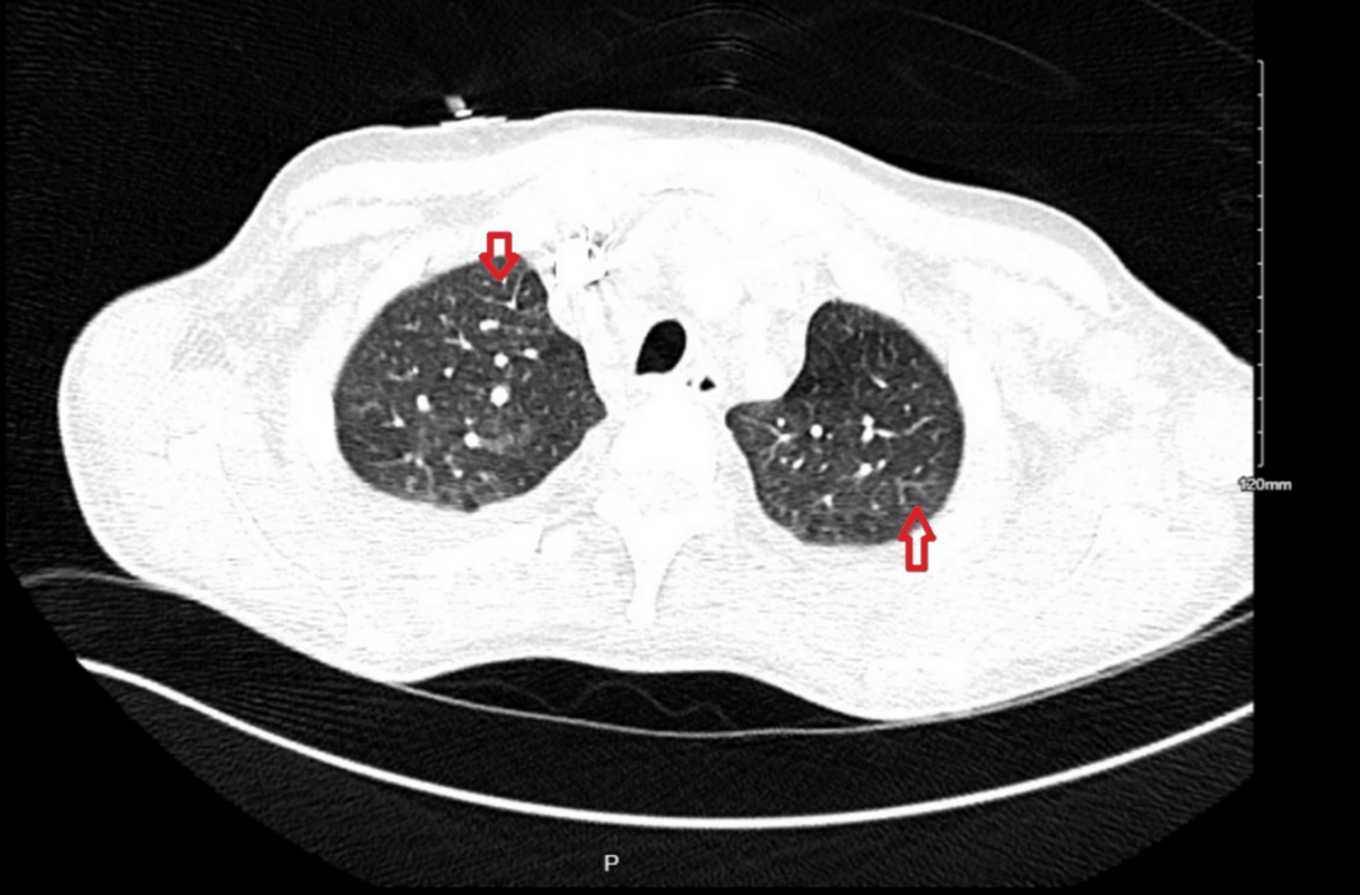
CT angio chest (axial view) Red arrows: tree-in-bud appearance in bilateral upper lung lobes CT, Computerized Tomography

**Figure 4:**
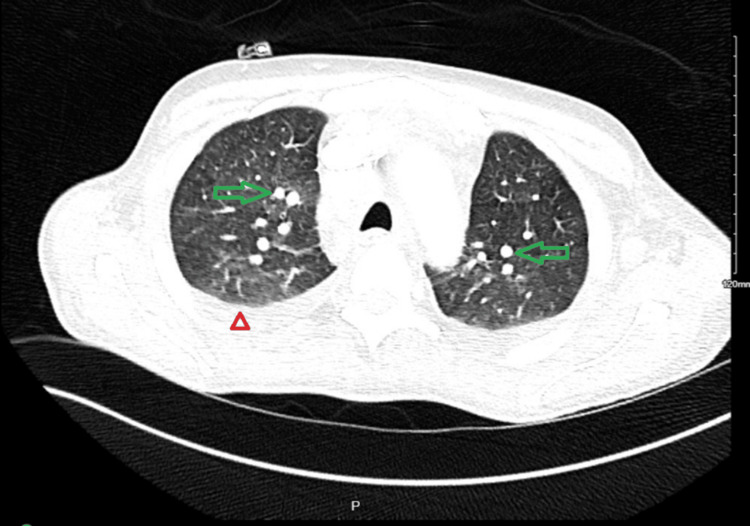
CT angio chest (axial view) Green arrows: lymph nodes; Red arrowhead: pleural effusion CT, Computerized Tomography

Initial laboratory tests were notable for leukocytosis, anemia, thrombocytopenia, kidney dysfunction, elevated liver enzymes, and metabolic acidosis (Table 1). Imaging of the chest and abdomen by computerized tomography (CT) revealed extensive lymphadenopathy, mild gallbladder wall thickening, and a right middle lobe opacity on chest X-ray (CXR). Magnetic resonance imaging/magnetic resonance cholangiopancreatography (MRI/MRCP) suggested cholecystitis (Figures 1-4).

**Table 1:** Initial laboratory and imaging results WBC, White Blood Cell Count; HIV, Human Immunodeficiency Virus; AST, Aspartate Aminotransferase; ALP, Alkaline Phosphatase; BUN, Blood Urea Nitrogen; eGFR, Estimated Glomerular Filtration Rate

Test	Result	Reference Values
WBC	20.92	4-11 x 10⁹/L
Hemoglobin	8.9	13-17 g/dL
Platelets	110	150-450 x 10⁹/L
CD4 Count	71	500-1,200 cells/mm³
HIV Viral Load	5,970,000	Undetectable
AST	165	<40 IU/L
ALP	370	40-129 IU/L
Lactate	3	<2 mmol/L
BUN	46	6-20 mg/dL
Creatinine	2.2	0.7-1.3 mg/dL
eGFR	36	>60 mL/min/1.73 m²
Venous Blood Gas	pH: 7.25, pCO₂: 46, Lactate: 3.0	pH: 7.35-7.45, pCO₂: 35-45 mmHg, Lactate: <2 mmol/L

The patient was initially started on empiric broad-spectrum antimicrobial therapy with vancomycin and piperacillin-tazobactam for sepsis coverage, fluconazole for oropharyngeal candidiasis, and Bactrim for *Pneumocystis jirovecii* pneumonia (PJP) prophylaxis. Additionally, the patient underwent a cholecystectomy for cholecystitis.

The differential diagnosis included lymphoma, disseminated MAC, and disseminated TB. Given these differentials, retroperitoneal lymph node and bone marrow biopsies were performed. Due to concern for disseminated MAC, empiric treatment with azithromycin and EMB was initiated, with levofloxacin avoided due to QTc prolongation. Subsequently, anti-tuberculous therapy was introduced with INH, EMB, and PZA, though RIF was deferred due to persistent transaminitis. After clinical stabilization, ART with Biktarvy was initiated.

Sputum cultures grew MAC; however, disseminated infection could not be initially confirmed. Bone marrow biopsy revealed multiple lymphohistiocytic aggregates with occasional granulomas, giant cells, and early necrosis. Lymph node biopsy demonstrated acid-fast bacilli in a background of necrotic tissue, with focal plasma cell, B-cell, and T-cell infiltrates - findings consistent with a mycobacterial infection. Unfortunately, lymph node cultures for microbiology were not available. Based on these results, the patient was continued on therapy for pulmonary MAC and possible disseminated MAC versus MTB, while awaiting definitive microbiological identification.

The treatment course was complicated by transaminitis and potential drug interactions. Eventually, MTB PCR from the lymph node biopsy returned positive for MTB, confirming the diagnosis of disseminated extrapulmonary TB. RIF was introduced once liver enzymes improved, but was later discontinued due to worsening transaminitis, prompting a switch to rifabutin. With this adjustment, liver function improved, and the patient remained clinically stable. The patient was discharged with a plan for continued outpatient follow-up.

## Discussion

Disseminated extrapulmonary TB and pulmonary *Mycobacterium* co-infection in newly diagnosed HIV patients present significant diagnostic and management challenges. HIV-induced immunosuppression increases susceptibility to both MTB and NTM, particularly MAC, leading to widespread dissemination and atypical presentations [8].

The distribution of extrapulmonary TB is strongly associated with HIV status, with lymphatic, disseminated, and meningeal TB occurring more often in HIV-positive individuals [15]. Declining CD4 counts increase the risk of extrapulmonary, rather than pulmonary, TB among HIV-infected individuals [16].

This highlights the need for early detection and differentiation between MTB and NTM infections, as standard TB treatments may not be effective against NTM species [17]. Diagnosing EPTB in HIV patients is challenging due to the paucibacillary nature of extrapulmonary infections and overlapping symptoms with other OIs [15]. The use of molecular diagnostic tools, such as GeneXpert MTB/RIF, has improved diagnostic accuracy, but culture-based confirmation remains necessary for distinguishing between MTB and NTM infections [8]. HIV-associated immunosuppression disrupts granuloma formation and compromises the host’s ability to contain mycobacterial infection, which accelerates dissemination [8]. This immune dysfunction results in rapid disease progression and a higher mortality rate among co-infected individuals [16]. ART initiation in HIV-TB co-infected patients improves survival but carries a significant risk of immune reconstitution inflammatory syndrome (IRIS), especially at low CD4 counts [18].

Concurrent TB and MAC infections in HIV pose considerable diagnostic difficulties because of their overlapping symptoms, atypical presentations, and limitations of available diagnostic tests [6,15]. Since both infections present with nonspecific systemic symptoms, including fever, weight loss, night sweats, and lymphadenopathy, distinguishing between them clinically can be challenging [13]. Although GeneXpert MTB/RIF enhances TB detection, it cannot identify MAC, making culture, despite its slow turnaround, necessary for diagnosis [8]. HIV-related immunosuppression alters typical radiographic patterns, so patients may lack cavitary lesions or even show a normal CXR, despite significant disease [9].

Additionally, disseminated MAC can mimic drug-resistant TB, delaying appropriate treatment if misdiagnosed [1]. In our case, the extensive lymphadenopathy, hepatosplenomegaly, and systemic symptoms necessitated a thorough diagnostic approach, including lymph node biopsy, bone marrow aspiration, and culture-based testing. However, given the delay in culture results, clinical expertise played a crucial role in guiding empiric therapy while awaiting definitive confirmation. This case underscores that, in the setting of HIV, where diagnostic tests may be inconclusive or delayed, clinical judgment remains indispensable in differentiating between MTB and MAC to ensure timely and appropriate treatment initiation.

Treatment of TB and MAC co-infection in HIV patients presents a significant therapeutic challenge due to overlapping drug toxicities, potential drug-drug interactions, and the prolonged duration of therapy required for both infections. Standard anti-TB therapy includes RIF, INH, PZA, and EMB for at least six months, whereas MAC treatment necessitates a macrolide (azithromycin or clarithromycin) in combination with EMB and RIF, often extending beyond 12 months [1,9,15,18]. In our case, treatment initiation was particularly challenging due to hepatotoxicity, which necessitated discontinuation of PZA. Additionally, the co-administration of RIF posed a challenge, as it reduces macrolide efficacy by inducing hepatic metabolism, potentially compromising MAC treatment. The patient was ultimately managed with INH and EMB for TB, alongside azithromycin and EMB for MAC, with rifabutin substituted for RIF to balance efficacy and minimize drug interactions. Another key challenge in dual therapy is adherence, as complex multidrug regimens increase the risk of missed doses and treatment failure. Furthermore, fluoroquinolones - often considered in resistant cases - were avoided due to concerns over QTc prolongation. This case highlights the necessity of a personalized treatment approach that accounts for drug tolerability, interactions, and patient-specific factors, emphasizing the importance of close monitoring to ensure both efficacy and safety in the management of these co-infections.

Managing concurrent HIV, TB, and MAC infection poses additional challenges, particularly regarding the timing of ART initiation. Evidence indicates that starting ART within two weeks of TB therapy improves survival in patients with CD4 <50, although it raises the risk of IRIS [18]. Delaying ART initiation reduces IRIS risk but leaves the patient vulnerable to further HIV-related immunosuppression. Our approach was guided by these recommendations: ART (Biktarvy) was initiated after clinical stabilization to minimize IRIS risk while ensuring immune recovery. The patient was closely monitored for IRIS, and supportive management strategies were in place. Although IRIS remains a concern, close monitoring and supportive care, including corticosteroids in severe cases, can mitigate complications [7]. Beyond IRIS, drug-drug interactions between ART and anti-mycobacterial therapies posed an additional challenge, requiring regimen adjustments to avoid subtherapeutic drug levels. Our case emphasizes the importance of balancing ART initiation timing to optimize immune restoration while managing potential inflammatory responses. Future research should focus on refining ART initiation strategies in the setting of multi-pathogen infections to enhance patient outcomes.

## Conclusions

This case illustrates the challenges in diagnosing and treating pulmonary MAC and disseminated extrapulmonary TB co-infection in an immunocompromised HIV patient. A comprehensive strategy integrating molecular diagnostics, clinical knowledge, and careful treatment selection is required due to overlapping clinical characteristics, delayed culture results, and drug-drug interactions. Effective management requires striking a cautious balance between reducing IRIS and restoring immunological function through early ART commencement, individualized treatment plans, and careful monitoring.
